# Health Disparity Resulting from the Effect of Built Environment on Temperature-Related Mortality in a Subtropical Urban Setting

**DOI:** 10.3390/ijerph19148506

**Published:** 2022-07-12

**Authors:** Zhe Huang, Emily Ying-Yang Chan, Chi-Shing Wong, Sida Liu, Benny Chung-Ying Zee

**Affiliations:** 1 Collaborating Centre for Oxford University and CUHK for Disaster and Medical Humanitarian Response (CCOUC), The Chinese University of Hong Kong, Hong Kong SAR, China; huangzhe@cuhk.edu.hk (Z.H.); cswong@cuhk.edu.hk (C.-S.W.); liusida2008@gmail.com (S.L.); 2 GX Foundation, Hong Kong SAR, China; 3 Centre for Clinical Research and Biostatistics (CCRB), The Chinese University of Hong Kong, Hong Kong SAR, China; bzee@cuhk.edu.hk; 4 Office of Research and Knowledge Transfer Services (ORKTS), The Chinese University of Hong Kong, Hong Kong SAR, China

**Keywords:** mortality, local temperature, green space, social and structural determinants of health disparities, Bayesian spatial analysis, Hong Kong

## Abstract

Whereas previous studies have assessed the overall health impact of temperature in Hong Kong, the aim of this study was to investigate whether the health impact is modified by local temperature of small geographic units, which may be related to the diverse socioeconomic characteristics of these units. The effects of local temperature on non-accidental and cause-specific mortality were analyzed using Bayesian spatial models at a small-area level, adjusting for potential confounders, i.e., area-level air pollutants, socioeconomic status, and green space, as well as spatial dependency. We found that a 10% increase in green space density was associated with an estimated 4.80% decrease in non-accidental mortality risk and a 5.75% decrease in cardiovascular disease mortality risk in Hong Kong, whereas variation in local annual temperature did not significantly contribute to mortality. We also found that the spatial variation of mortality within this city could be explained by the geographic distribution of green space and socioeconomic factors rather than local temperature or air pollution. The findings and methodology of this study may help to further understanding and investigation of social and structural determinants of health disparities, particularly place-based built environment across class-based small geographic units in a city, taking into account the intersection of multiple factors from individual to population levels.

## 1. Introduction

Whereas climate change has become a widely recognized global challenge in this century, its human health impacts have wide implications. As a result of the increasing concern regarding the public health effects of meteorological conditions, such as extreme temperatures, associations between extreme temperatures and mortality/morbidity have been identified repeatedly and consistently in studies from around the world. A substantial number of existing studies examined associations between meteorological variables and health-related outcomes using daily time series study design [1,2,3,4,5]. Findings suggest that both high and low temperatures increase mortality, with heat-related mortality occurring during the first few days after temperature increase, whereas the effects of coldness are prolonged for several weeks [6,7,8]. Apart from all-cause or non-external causes of mortality, cardiovascular and respiratory deaths have been found to be associated with both heat and cold exposure [4,9,10,11,12], with V-, U-, or J-shaped temperature–mortality relationships observed, with the optimum temperature corresponding to the lowest point in the curve. Other causes, including cancer, have been shown to have a substantially weaker association with ambient temperatures [6,13].

Such health studies have measured ambient temperature exposures of entire study populations using city- or country-wide measurements from either one monitoring station or the average of a network of stations, which generally does not account for within-city spatial variation. This failure to account for within-city spatial variations may result in decreased statistical power and measurement error, as temperatures vary across a city, in addition to overlooking the possibility of exploring place-based temperature-related health disparities. Moreover, previous studies examining the association between air pollution exposure and health using different interpolation methods from the measurements of nearby monitors suggest that within-city effects are larger than between-city effects [14,15]. Nevertheless, given the limited knowledge about the explanatory power of meteorological and air quality (MAQ) exposures at the geographic subunit level, further investigation is needed with respect to the geographic heterogeneity of the relationship between temperature and mortality within a city, which could be achieved appropriately by using small-area-level ecological designs.

In contrast to time-series studies, small-area-level ecological studies offer another means of examining the combined effects of acute and chronic environmental exposure on health [16] to identify subgroups at high risk within a region by exploring the spatial distribution of health risk and therefore place-based health disparity. Mapping the spatial variations of health risk from environmental exposure is useful for improving public health intervention resource allocation to alleviate health disparity. However, some previous ecological studies investigating the pattern of mortality did not take into account how areas with different characteristics were distributed in the study region. Thus, the influence of a small area was not considered to depend on its location [17,18]. The same estimates would be obtained if the distribution of small areas were permutated at random [19]. Meanwhile, ignoring the spatial dependency of data may lead to underestimated standard errors on regression coefficients and result in an incorrect conclusion, as adjacent areas in a region tend to have more similar characteristics than disconnected areas. Subsequently, the incorporation of spatial dependency in a model could improve the estimation of health risk in an area [20]. With spatially referenced public health data becoming more available, statistical methodology for the analysis of spatial patterns in disease mapping has been developed rapidly to facilitate investigations of disease occurrence or mortality [21,22,23], accounting for spatial dependency. Areal or lattice data perspectives have been used to model regional mortality for the study of discrete spatial units [15,24,25], whereby spatial dependency is taken into account through the spatial weight matrix.

Besides air pollutants, socioeconomic status and green spacing, as social and structural determinants of health, could confound the effect of temperature on health. First, the evidence that air pollutants affect health is tremendous. For example, the effects of particulate matter (PM) on mortality have been well-recognized in both the short and long term [26,27]; NO_2_ and O_3_ are positively associated with health outcomes [28,29]. Hence, air pollutants are often included as confounders in studies examining the relationship between temperature and health and vice versa [30,31,32]. Secondly, previous studies revealed that the differences in mortality risk across various areas could be partially attributed to differences in demographic characteristics [33,34,35]. Furthermore, how socioeconomic disparities account for variations in health outcomes between societies has also been investigated in previous studies [36,37]. Finally, a considerable amount of attention has been given to green space because it not only supports the ecological integrity of a city but also promotes physical activity and improves health [38,39,40]; recreational walking was found to explain the relationship between green space and physical health [41].

In order to synthesize the spatial variations of exposures, spatial dependency, and potential confounders, Bayesian spatial models were constructed in this study to examine the citywide temperature–mortality association in small areas in Hong Kong. The aim of the study is to investigate whether neighborhoods with high temperatures coincide with reduced or increased levels of mortality, accommodating random spatial effects and covariate effects (i.e., other meteorological factors, air pollutants, socioeconomic characteristics, and green space).

Under the Health-Emergency and Disaster Risk Management (Health-EDRM), an emerging academic paradigm adopted by the World Health Organization, health and disaster risks are examined through public health tools for the management of health and disaster risk [42]. Hence, climate change and ensuing extreme temperatures, as major disaster risk drivers, as well as their effect on health, should be considered within the disaster risk reduction context. However, little is known about the effect of extreme temperatures on health at the small-area level, potentially leading place-based health disparity. This study is motivated by the need to ascertain regional variations and disparity with respect to mortality and assess the potential social and structural sources of these variations. Relevant findings will provide evidence to further support the Health-EDRM Framework, linking health and disaster risk, and address health disparity in urban setting [43].

## 2. Materials and Methods

This is an aggregated ecological study analyzing secondary data collected by government authorities in Hong Kong. Hong Kong is a city in southern China and home to more than seven million inhabitants, with a subtropical climate. Hong Kong has a warm and humid spring, hot and rainy summer, cool and dry autumn, and cold and almost rainless winter. Its average annual temperature is 23.5 °C, and the average annual rainfall is 2431.2 mm, with high humidity in March and April. The temperature in central Hong Kong sometimes drops below 10 °C, whereas the temperature in the inland New Territories and highlands can drop below 0 °C with frost [44]. Hong Kong is also a densely populated vertical city affected by urban heat island effects with a complex spatial distribution of population and severe income disparity. In this study, analyses were performed at the tertiary planning unit (TPU) level, which is the smallest geographical area level for which data on mortality and socioeconomic characteristics are available from the Hong Kong Special Administrative Region (SAR) Government.

### 2.1. Data Collection

**Figure 1:**
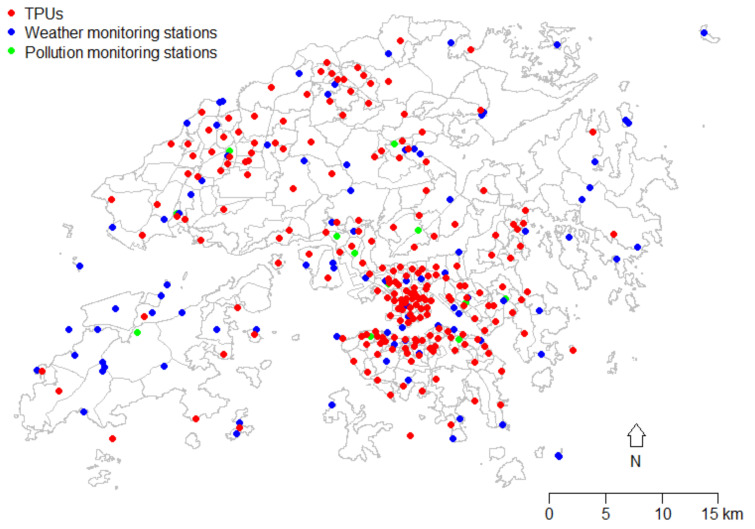
Locations of TPU centroids, weather, and air quality monitoring stations.

This study involved a range of meteorological, pollution, socioeconomic, geographic, green space, and mortality data at the small-area level, where socioeconomic data are only available in census years. Due to the boundary changes in each census and the limited information collected in previous censuses, all analyses in this study were based on geographic and socioeconomic data from 2016, as this year has the greatest number and completeness of data. In 2016, there were 291 TPUs in Hong Kong, but some adjacent TPUs were merged to protect data privacy when the government released the statistics. Hence, the whole land area of Hong Kong in this study is divided into 214 subregions. Figure 1 shows the centroids of the 214 subregions.

#### 2.1.1. Mortality Data

For the year 2016, all non-accidental mortality (codes A00-R99 of the International Classification of Diseases, version 10 (ICD-10)) and cause-specific mortality due to cancers (C00-D48), respiratory diseases (J00-J99), and cardiovascular diseases (I00-I99) were obtained for each subregion from the Census and Statistics Department of the Hong Kong SAR Government.

#### 2.1.2. Socioeconomic Data

Because socioeconomic covariates typically correlate, we performed principal component analysis on 27 variables related to socioeconomic vulnerability in Hong Kong. In other words, the intersection of multiple factors from individual to population levels was taken into account. The five principal components (PC) accounted for more than 77% of the variation, namely: (1) indigenous degree: the lack of access to international information; (2) family resilience: the status of lack of family support; (3) individual productivity: an individual’s education, occupation, and income in the subregion; (4) populous grassroots: the situation of lack of home ownership; and (5) young age: youthfulness. The representativeness and detailed method of construction of the five principal components in the setting of the Hong Kong population were previously reported in [45].

#### 2.1.3. Meteorological and Pollution Data

Daily data on the minimum temperature at all weather stations from the Hong Kong Observatory were extracted, excluding data from stations with an altitude higher than 200 m, as they are not representative of the population, who do not actually reside at such a high altitude; data from alternative nearby stations were used instead. For this study, we decided to consider only minimum temperature because previous studies revealed that models using various air temperature measures (mean, minimum, and maximum) were fairly similar in terms of predicting mortality [46,47]. Moreover, using minimum temperature from nearby monitoring stations may be more appropriate to estimate people’s night-time temperature exposure, as census and mortality data assign people to a TPU according to their residential addresses, and people are more likely to stay home at night. Other spatially referenced meteorological data, including daily relative humidity, rainfall, and dew point temperature, from various stations were also obtained from the Hong Kong Observatory.

Daily pollution data, including levels of PM_2_._5_, NO_2_, O_3_, PM_10_, and SO_2_, for all 13 general air quality monitoring stations in Hong Kong were obtained from the Environmental Protection Department of the Hong Kong SAR Government for model adjustment.

The annual average temperature in all the subregions studied in the year 2016 was the primary exposure measure of interest due to the increased network of government monitors in place in previous years. The locations of the monitoring stations in 2016 are indicated in Figure 1. Stations with the missing values exceeding 25% during the study period were excluded. Daily data on exposure variables for each station (including weather and air quality stations) were averaged to obtain the annual averages in 2016. Based on the location of a subregion’s centroid, the nearest weather and air quality monitoring stations were identified and used to assign local exposure estimates to each sub-region [48]; the median distance from each subregion centroid to the nearest temperature station was 1.63 km (ranging from 0.05 to 10.51 km).

#### 2.1.4. Green Space Data

To measure green space coverage, we obtained green space data from the 2018 Land Utilization in Hong Kong document [49]. These data are a rough presentation of the distribution of land uses in Hong Kong, mainly compiled by the Planning Department of the Hong Kong SAR Government using satellite images and in-house survey. The raster grids have a spatial resolution of 10 m. In this study, green space is defined as woodland, shrubland, grassland, or wetland. Green space forms part of the human-made space of a city in which people live, work, and recreate on a day-to-day basis and can therefore be considered part of the built environment. Green space density is derived from a ratio of the area of green space to the whole area for each subregion using ArcGIS software. Because green space density data were not available for 2016, 2018 data were used as a proxy, as green space coverage does not change dramatically across a short space of time.

### 2.2. Statistical Methods

Spatial autocorrelation is common in areal data. To test for the presence of spatial autocorrelation of mortality risk, Moran’s I tests were used to detect clusters or hotspots [19] before subsequent analysis. If spatial autocorrelation were found, the associations between temperature and mortality would be assessed through a generalized linear mixed model (GLMM) with a Bayesian approach, including random effects with prior distributions describing the spatial dependency.

The Besag–York–Mollié (BYM) model [50], a commonly used Bayesian disease-mapping method, was employed to model the case counts of mortality, assuming that the health risk of a subregion is affected by its immediate neighbors (i.e., subregions that share a common border) with equal weight. As the case counts of mortality tend to be over-dispersed, they are often modelled as a negative binomial distribution to avoid underestimation of the standard errors. Because the whole land area of Hong Kong is made up of hundreds of islands, for the five TPUs made up of islands with no immediate adjacent neighbors, additional links were added based on road networks or ferry connections to reflect the “true” connectedness structure of Hong Kong [36].

To estimate the effect of local exposures on mortality, the relative risk (RR) and the corresponding 95% credible intervals (95% CI) were calculated after adjusting for the confounding effects of area-level socioeconomic, pollution, green space, and other meteorological variables. The model estimates area relative risk based on a negative binomial regression as follows:

*Y_i_* ∼ *NB*(*μ_i_*, *α*)

*log(μ_i_)* = *β_0_* + *X_Ai_β_A_* + *log(population_i_)* + *u_i_* + *v_i_*

$$u_{i}∼\;iCAR({\bar{u}}_{i},\;\sigma_{u}^{2}/n_{i})$$

$$v_{i}∼\;N(0,\;\sigma_{v}^{2})$$

where the annual case count *Y_i_*, is drawn from a negative binomial distribution, with mean *μ_i_* and shape parameter α; log(*μ_i_*) is the linear predictor, including a global intercept *β_0_*, regression coefficients *β_A_*, the area-level covariates *X_A_* (i.e., meteorological, pollution, green space density, and socioeconomic variables), an offset term *log(population_i_)*, and random effects *v_i_* and *u_i_*. An offset term is included as a correction factor to model the mortality rate because the higher the population, the higher the mortality. In addition, *u_i_* is the spatially structured random effect using the intrinsic conditional autoregressive (iCAR) prior, where *n_i_* indicates the number of neighbors, and *v_i_* is the spatially unstructured random effect using an exchangeability prior. It should be noted that vague priors are assumed, for *α* and all *σ*^2^s, to have both large mean and variance, and *β*s are assumed to have zero mean with large variance [51].

Non-accidental mortality and cause-specific mortality of cancers, respiratory diseases, and cardiovascular diseases were used as outcome variables. For each outcome, univariable models were fitted, including the intercept, offset term, and random effects, with one of the area-level covariates studied at a time; then, multivariable model fitting was conducted with all area-level covariates significant in the corresponding univariable model, which was reflected by the 95% CI of the estimate for the regression coefficient, not including 0. The model fit was evaluated by the deviance information criterion (DIC) [52].

Sensitivity analyses were used to compare the main findings using meteorological and pollution data from 2016 with the corresponding data from 2015. R software version 3.6.1 (R Foundation for Statistical Computing, Vienna, Austria) [53] was used for data analysis. The spdep and DCluster packages were used to detect spatial autocorrelation, maptools package was used to create maps, and INLA package was used to implement Bayesian estimation. It should be noted that the Integrated Nested Laplace Approximation (INLA) [54] was used as a less computationally intensive alternative to Markov Chain Monte Carlo sampling for Bayesian inference. Ethical approval for the study was obtained from the Survey and Behavioural Research Ethics Board of the university sponsoring the study.

## 3. Results

### 3.1. Spatial Patterns of Mortality and Temperature

**Figure 2:**
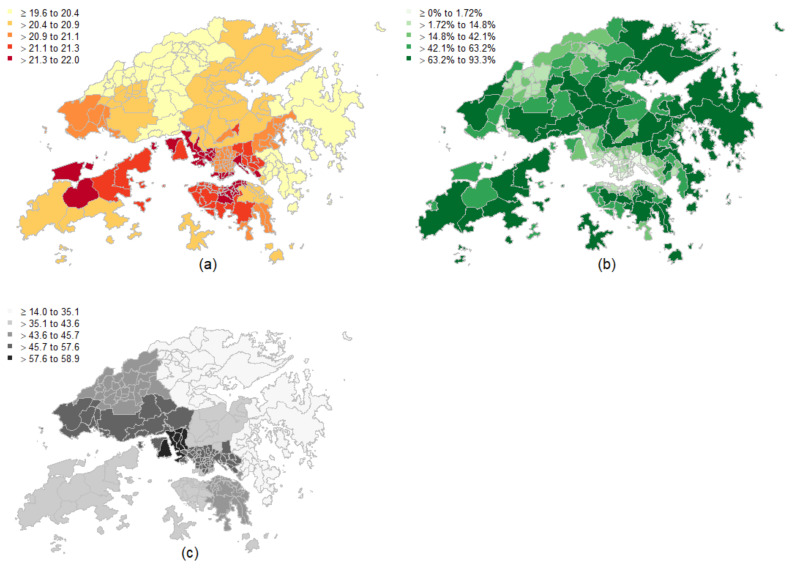
Environmental exposures in Hong Kong in 2016. Note: (**a**) minimum temperature (°C); (**b**) green space density (%); (**c**) NO_2_ (µg/m^3^).

**Figure 3:**
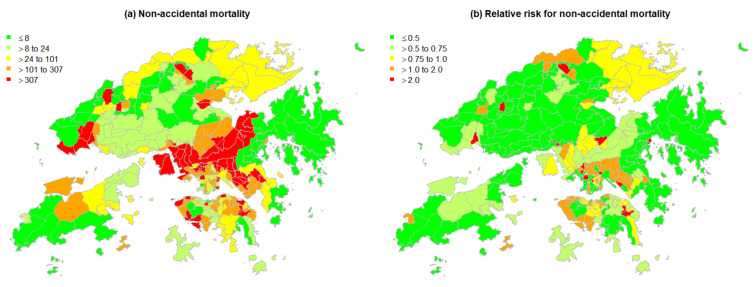
Non-accidental mortality and crude relative risk of mortality in Hong Kong in 2016. Note: a five-level scale is used to provide a balance between differentiation and clarity.

Table 1 presents a statistical description of the socioeconomic variables and environmental exposures tested. The five socioeconomic variables are the five principal components of socioeconomic vulnerability derived from Huang et al. [45]. The annually averaged minimum temperatures (°C) in different small areas of Hong Kong ranged from 19.6 to 22.0. Figure 2 shows maps of the geographical distributions of some environmental exposures. During the study period, there were 44,543 non-accidental mortality cases in Hong Kong, with the central parts of Hong Kong shown to be clusters of high mortality among all subregions, whereas areas of low mortality were located in the periphery of the city (Figure 3a). Figure 3b maps the crude relative risk for mortality of all subregions compared to the whole of Hong Kong.

**Figure 4:**
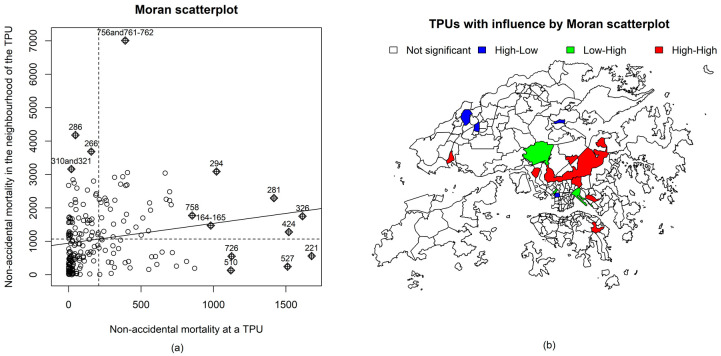
Spatial autocorrelation of non-accidental mortality in Hong Kong. Note: (**a**) the x-axis represents values in area i, whereas the y-axis represents the spatially weighted sums of values in the neighborhood of location i. Diamond-shaped points are areas whose local relationships influenced the slope of the straight line more than proportionately; the numbers above the diamonds are their TPU IDs. (**b**) High-High indicates that the mortality of the area and the average of its neighbors were higher than the global mean; High-Low indicates that the mortality of the area was higher than the global mean but that the average mortality of its neighbors was lower than the mean (Low-High indicates the inverse).

Global Moran’s I indicates that the spatial patterning of non-accidental mortality was significant (Moran’s I = 0.11, *p*-value < 0.01), with the solid diagonal line indicating the presence of positive spatial autocorrelation throughout all areas (Figure 4a). Areas with a significant influence on the slope of the straight line, as in a linear model, were located. Figure 4b presents the areas with significant influence by Moran scatterplot quadrant.

**Figure 5:**
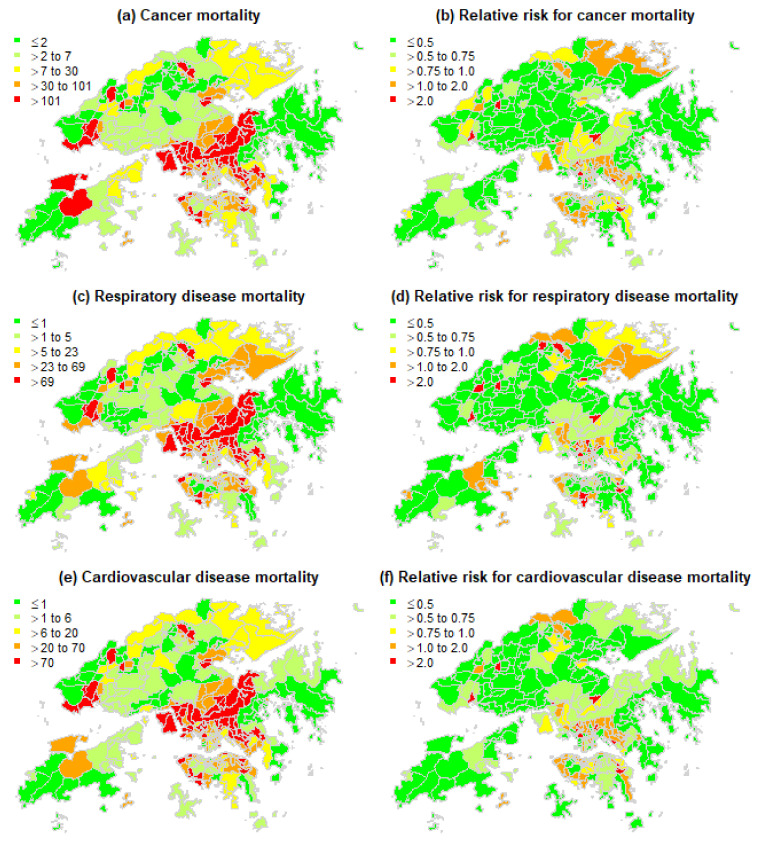
Cancer, respiratory disease, and cardiovascular disease mortalities and crude relative risk of mortality in Hong Kong in 2016.

In addition, among all types of non-accidental deaths, the most common was cancers, which accounted for 31.8% of non-accidental mortality. This was followed by respiratory diseases (24.0%) and cardiovascular diseases (22.4%). Some cause-specific mortality maps are displayed in Figure 5.

### 3.2. Associations of Mortality and Temperature

To summarize the fitted models, Table 2 presents the posterior mean of the regression coefficients and 95% CI for each of the covariates in the model. All meteorological, pollution, socioeconomic, and green space variables, as well as minimum temperatures of interest, were included in the BYM models to estimate their effects on mortality.

Because the shape parameter α from all the negative binomial regression models was large, overdispersion might not be a problem here. However, because the DIC of the negative binomial model was much smaller than that of the corresponding Poisson model, negative binomial models were preferred in this study. In addition, the difference of DIC (∆DIC) presented in Table 2 indicates that the socioeconomic variables and environmental exposures did explain part of the variability in the risk of all four types of mortality. To test the significance of the spatial effect on mortality, the same models were constructed, but the random effects in the linear predictor were excluded. The resulting DICs of these new models were generally larger than the original DICs (not shown in table), suggesting the presence of random effects.

In univariable models, populous grassroots and young age were positively and negatively correlated with all four types of mortality, respectively, which means higher populous grassroots or lower young age scores indicate higher mortality from non-accidental, cancer, respiratory, and cardiovascular causes after accounting spatially structured and unstructured random effects. In addition, NO_2_ and minimum temperature appeared to explain, to some extent, the variation in the four types of mortality. However, in multivariable models, inclusion of neither temperature nor NO_2_ significantly affected the four types of mortality, with the 95% CIs straddling zero. In addition, except for respiratory disease mortality, the regression coefficients of populous grassroots and young age were not considerably modified by the presence of the other covariates, although the coefficients of green space density changed substantially in the multivariable models with the expected sign. Sensitivity analyses showed consistent results in terms of the direction and magnitude of the regression coefficients but with slightly stronger effects of green space density.

#### 3.2.1. Non-Accidental Mortality

Initial univariable models show that apart from populous grassroots, young age, and green space density, both annual estimates of NO_2_ and minimum temperature were significantly and positively associated with non-accidental mortality, with a relative risk (RR) of 1.02 (95% CI: 1.01–1.05) for NO_2_ and 1.75 (95% CI: 1.23–2.50) for minimum temperature. When green space density and socioeconomic variables were added in the multivariable model, the intensity of both associations was reduced toward zero. The results show that an increase of 10% in green space density was associated with a decrease of 4.8% (1 − exp(−0.0492)) in the risk of non-accidental mortality rate, with a 95% CI ranging from 0.4% to 9.0%. The pattern of all-cause mortality was similar to that of non-accidental mortality (not shown in table), constituting 96.3% of all death cases.

**Figure 6:**
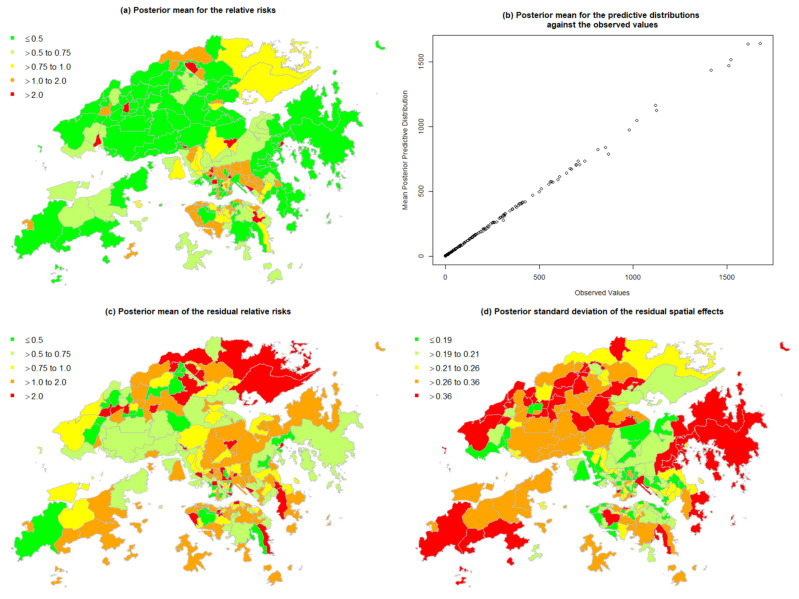
Posterior means of the relative risks for non-accidental mortality obtained with the multivariable model using INLA.

In comparison with Figure 3b, Figure 6a seems to produce similar estimates in all the small areas. Figure 6b indicates that on average, the prediction is close to the observed values. Figure 6c shows a map of the posterior mean of residual relative risk of the mortality for each subregion compared to that of the whole study region after the risk factors were taken into consideration. Regarding uncertainty, Figure 6d shows a posterior standard deviation map of the spatial effect on mortality, where the peripheral city subregions tend to have a more variable RR of non-accidental mortality than those in the central region of Hong Kong.

#### 3.2.2. Cancer Mortality

Apart from family resilience, the results of univariable models for cancer were consistent with non-accidental mortality, although with slightly reduced effect sizes for all covariates. The association with green space density (RR: 0.46, 95% CI: 0.28–0.75) was the weakest among the four types of mortality studied, and the significant association in the univariable model became non-significant in the multivariable analysis. In addition, the coefficients of both socioeconomic variables (populous grassroots and young age) were similar to those obtained from univariable analysis.

#### 3.2.3. Respiratory Disease Mortality

For univariable models, indigenous degree, populous grassroots, NO_2_, and minimum temperature were positively correlated, whereas family resilience, individual productivity, young age, and green space density were negatively associated with respiratory disease mortality. When all these variables were considered simultaneously, variables of indigenous degree, family resilience, NO_2_, and minimum temperature became non-significant, with the corresponding regression coefficients dropping substantially; however, NO_2_ was close to significant at the 95% credible level (RR: 1.01, 95% CI: 1.00–1.03). In addition, the effect of populous grassroots (RR: 1.54, 95% CI: 1.31–1.81) seemed to decrease when compared with the univariable model (RR: 1.41, 95% CI: 1.21–1.66). The significant covariate of individual productivity suggests that areas with a higher individual productivity score had lower respiratory disease mortality.

#### 3.2.4. Cardiovascular Disease Mortality

The observed associations for cardiovascular disease mortality closely followed the pattern for non-accidental mortality in both univariable and multivariable models. After entering all the significant variables into the same model, we expected a 5.75% (=1 − exp(−0.0592)) decrease in the risk of cardiovascular disease mortality in association with a 10% increase in green space density in a subregion, assuming all other predictors were fixed, i.e., the higher the percentages of green space in a neighborhood, the lower the cardiovascular disease mortality.

## 4. Discussion

In this study, we analyzed the effect of local temperature on mortality, adjusting for potential confounders, i.e., area-level air pollutants, socioeconomic status, and green space, as well as spatial structure. We found that a 10% increase in green space density was associated with an estimated 4.8% decrease in non-accidental mortality risk, but the spatial variation of annual temperature did not significantly contribute to mortality.

Further investigations of the multivariable models indicated that an area-specific effect contributed substantially to the model fits. It should be noted that the estimates of the residual RRs for non-accidental mortality shown in Figure 6c were higher, on average, in populous or less developed areas. The higher variability of the area-specific effect in the city periphery may be due to the lack of contiguous areas. Increased-risk small areas were also identified; these clusters of neighborhoods with high mortality (High-High) were located in the central part of Hong Kong (Figure 4b) and categorized as “suburban areas” in a previous study [45].

The significant effects of green space density and socioeconomic variables in the four models demonstrate how social and structural determinants alleviated or exacerbated the RR for the four types of mortality. The differences in DIC for the four types of mortality shown in Table 2 suggest that green space density and socioeconomic variables explain part of the variability in risk of mortality, which was not taken into account in many time series models but can confound the relationship between temperature and mortality.

In this study, spatial annual non-accidental mortality was significantly associated with annual temperature and NO_2_ in univariable models after taking into account random effects at the small-area level. These results are consistent with those reported by Huang et al. [55], who found that areas with lower socioeconomic status and higher annual temperature had generally higher mortality in 66 communities in mainland China, as well as a previous ecological study, which revealed that an excessive number of people died from non-external causes and circulatory and respiratory causes attributable to annual average NO_2_ concentration in Auckland [56]. The underlying mechanisms of increased mortality associated with elevated temperature may be linked to the stress of high temperature placed on the respiratory and circulatory systems, which may increase cardiorespiratory death risk [57]. In addition, the health effects of air pollution can be explained by oxidative stress and immune system damage after both short-term and long-term exposures [58].

However, the multivariable models suggest that annual temperature and NO_2_ had little effect on any if the four types of mortality studied after adjusting for neighborhood green space density and socioeconomic variables. One plausible explanation is that these variables were significantly correlated with each other, as shown in Table 1. Thus, the presence of green space density modified and shrank the coefficients of the temperature and NO_2_ toward zero. Another possible reason may lie in the relatively small spatial variability of the temperature and NO_2_ concentrations in this study when compared with green space data. Therefore, most effects on mortality were attributed to green space density. It should be noted that green space data were effectively time-invariant on a daily basis but varied substantially between small areas, which is suitable for ecological study, whereas meteorological and pollution data were more time-varying than space-varying on a daily basis for the whole area across the study region. This is the likely reason why temperature and NO_2_ were significantly associated with mortality in previous time series studies but not here. Further studies are needed to obtain larger spatial variability by generating a continuous surface for the variable of temperature or NO_2_ concentration using geostatistical models (e.g., kriging) [59,60,61] and to disentangle the mixture effects of meteorological factors, air pollutants, and green space to which people are exposed.

The mechanisms of the beneficial effects of green spaces in urban environments include that they alleviate extreme temperature resulting from urban heat island effects, air pollution, and ambient noise, in addition to promoting healthy behaviors (e.g., physical activity and social interaction relatively lacking in urban environment) that play an important role in physical and psychosocial well-being. With this study, we found that living in areas with high green space density was associated with a lower risk of non-accidental mortality, especially for cardiovascular and respiratory diseases but not with cancer, which is consistent with the results of previous studies. Gascon et al. [62] found that living in areas with higher amounts of green space reduced cardiovascular mortality. Mitchell and Popham [40] indicated that the association between income deprivation and mortality was modified by exposure to green space but not for cancer. In Hong Kong, Wang and co-authors suggested that a 10% increase in coverage of green space was associated with decreased all-cause mortality (hazard ratio: 0.963, 95% CI: 0.930–0.998) among the elderly [63], which is similar to our findings (RR: 0.952, 95% CI: 0.910–0.996). Xu et al. showed that Hong Kong residents living in the greenest areas had a lower risk of cardiovascular mortality, whereas lung cancer mortality had no significant association with green space [64]. In addition, the non-significant association between cancer mortality and green space in this study may also suggest that cancers are less likely to be caused by environmental than lifestyle factors.

Given the links between green space as part of the built environment and health, bringing green space to various small areas of the city was increasingly valued during the process of urbanization, which may also help alleviate health disparity across small areas. as the findings of this study suggest. However, the literature suggested that only those with a high level of education or high income benefited from urban green space [65], since urban greening might lead to increased property rent and housing prices, which could encourage the displacement of the deprived population [66,67]. Because socioeconomic deprivation is usually geographically correlated with less exposure to green space, proactive green space interventions by the government are likely to be successful in reducing the mortality risk related to adverse environmental exposure in socioeconomically vulnerable communities, thereby reducing health disparity arising from social and structural determinants of health. In view of the worsening extreme temperature scenario due to the climate crisis, we hope that the findings of this study can help to persuade the government to adopt a policy of modifying the urban built environment by providing more green space for socioeconomically deprived areas in future urban planning and design. In addition, site selection for future government housing construction for those most socioeconomically deprived could take the existing surrounding green space into consideration to reduce health disparity.

Using 2006 data in Hong Kong, through generalized additive mixed models, Thach et al. found that spatially referenced physiological equivalent temperature (PET) was significantly associated with mortality, with PET as a compound variable, taking air temperature and greenery of the surroundings into account [68]. They suggested that improvement of greenery coverage could be an effective intervention for reducing PET-related mortality. In this study, significant associations between green space density and different types of mortality rate were also observed, and these findings became more evident when similar results were obtained with different approaches.

### Strengths and Limitations

Hong Kong offers a good opportunity to study social and structural determinant-related health disparity issues due to the high level of income inequality and the well-recorded and accessible government data, with socioeconomic, green space, meteorological and pollution data freely available from government. The greatest strength of this study lies in the consideration of socioeconomic information and green space density as social and structural determinants of health disparity, combined with the full use of the monitoring network for local meteorological and pollution data to model mortality in a subtropical Chinese city. In addition, land use data for green space used in this study, as an official record from the government, is relevant to prompting policy changes to reduce place-based health disparity.

Limitations of this study include the ecological fallacy, as there could be discrepancies between individual-level and area-level associations between the same exposure and health outcomes. Future studies might consider supplementing individual-level data to integrate the results at different levels within the same city. Another limitation is that although annual averages of local meteorological and pollution data used in this study concerned spatial variability, they failed to capture the temporal variation on a daily basis. Further study could focus on a spatiotemporal study design using daily meteorological and pollution data. Thirdly, the accessibility and quality of green spaces were not considered, based on the assumption that the effects of all vegetation are the same. Future research should challenge this assumption by differentiating types of green space according to their characteristics and accessibility.

## 5. Conclusions

Previous studies indicated that the daily fluctuation in mortality was associated with daily temperature when a whole territory was taken as spatially uniform, whereas in this study, we found that the spatial variation of mortality (i.e., place-based health disparity) within the study region can be explained by the geographical distribution of green space and socioeconomic factors as social and structural determinants, controlling for temperature variations across the territory. We observed a protective relationship between green space density, as part of the built environment in an urban city, and the risk of non-accidental mortality, cardiovascular disease mortality, and respiratory disease mortality but not cancer mortality. Although there was no apparent evidence showing that the use of local temperature improves the assessment of the temperature–mortality association, this study extends the knowledge about the confounding effect of green space. Given that high coverage of green space was associated with a decrease in a range of mortalities, future studies should further investigate the mechanism of these associations so that policy makers can modify the urban built environment via urban planning and design to mitigate the adverse effects of environmental exposure on health, as well as place-based health disparity in terms of temperature-related mortality, particularly when extreme temperatures and their threat to health could become more frequent and severe with the progressing climate crisis.

## Figures and Tables

**Table 1:** (a) and (b): Descriptive statistics and Pearson correlations of socioeconomic variables and environmental exposures in Hong Kong in 2016.

(a) Descriptive Statistics															
Area-Level Characteristic					Minimum		25th Percentile			Median		75th Percentile		Maximum	
Indigenous degree score					−4.5		−0.4			0.3		0.6		1.2	
Family resilience score					−4.2		−0.4			0.2		0.5		2.4	
Individual productivity score					−3		−0.6			0.1		0.6		3.1	
Populous grassroots score					−2.8		−0.8			0		0.8		2.6	
Young-age score					−2.4		−0.6			−0.2		0.5		5.4	
PM_2_._5_ (µg/m^3^)					18.5		20.0			21.5		22.9		27.0	
NO_2_ (µg/m^3^)					14.0		37.5			45.6		56.5		58.9	
O_3_ (µg/m^3^)					32.0		33.0			41.1		43.5		62.5	
PM_10_ (µg/m^3^)					28.4		30.6			32.0		34.3		43.7	
SO_2_ (µg/m^3^)					4.6		6.1			9.3		9.8		11.6	
Minimum temperature (°C)					19.6		20.6			21.0		21.3		22.0	
Rain (mm)					2158		2579			2857		3033		3464	
Dew temperature (°C)					18.8		19.1			19.4		19.8		20.3	
Relative humidity (%)					72.9		78.9			80.5		81.6		85.5	
Green space density (%)					0.0		3.4			32.4		57.0		93.3	
**(b) Pearson Correlations**															
	**PC 1**	**PC 2**	**PC 3**	**PC 4**	**PC 5**	**PM_2_._5_**	**NO_2_**	**O_3_**	**PM_10_**	**SO_2_**	**min.T**	**Rain**	**dew.T**	**RH**	**GS**
PC 1	1														
PC 2	0	1													
PC 3	0	0	1												
PC 4	0	0	0	1											
PC 5	0	0	0	0	1										
PM_2_._5_	0.2	−0.08	−0.1	0.02	0.07	1									
NO_2_	0.11	−0.1	0.15	0.2	−0.06	0.6	1								
O_3_	−0.23	0.06	0.07	0.02	−0.03	−0.77	−0.75	1							
PM_10_	0.18	−0.07	−0.09	0	0.14	0.91	0.5	−0.55	1						
SO_2_	0.02	−0.14	−0.01	−0.01	0	0.66	0.54	−0.71	0.59	1					
min.T	−0.28	−0.26	0.3	0.34	−0.17	0.04	0.39	0.01	0.06	0.05	1				
Rain	−0.05	0.03	0.27	0.15	−0.11	−0.36	−0.05	0.22	−0.45	−0.44	0.18	1			
dew.T	−0.16	−0.29	0.13	0	−0.14	−0.25	−0.27	0.44	−0.1	−0.17	0.2	−0.09	1		
RH	0.08	−0.01	−0.06	−0.07	0.01	−0.34	−0.41	0.44	−0.18	−0.28	−0.37	−0.01	0.64	1	
GS	−0.2	0.37	−0.21	−0.11	0.07	−0.29	−0.41	0.31	−0.22	−0.1	−0.31	−0.17	0.06	0.28	1

Note: The table cells shaded in grey indicate that the *p*-value of the corresponding correlation coefficient was less than 0.05. PC 1: indigenous degree score; PC 2: family resilience score; PC 3: individual productivity score; PC 4: populous grassroots score; PC 5: young-age score; min.T: minimum temperature; dew.T: dew temperature; RH: relative humidity; GS: green space density.

**Table 2:** Summary statistics for the posterior mean and 95% credible interval for the regression coefficients of the negative binomial regression models with respect to the four types of mortality.

	Non-Accidental Mortality (44,543 Cases)						Cancer Mortality (14,175 Cases)					
	Univariable			Multivariable (∆DIC = −38.0)			Univariable			Multivariable (∆DIC = −47.2)		
	Mean	Lower	Upper	Mean	Lower	Upper	Mean	Lower	Upper	Mean	Lower	Upper
Intercept				−6.756	−12.788	−0.748				−8.04	−13.781	−2.339
Indigenous degree	0.104	−0.046	0.254				0.124	−0.015	0.262			
Family resilience	−0.158	−0.319	0.002				−0.167	−0.314	−0.020	−0.084	−0.213	0.045
Individual productivity	−0.109	−0.266	0.048				−0.101	−0.245	0.045			
Populous grassroots	0.487	0.350	0.626	0.443	0.313	0.757	0.468	0.340	0.599	0.437	0.314	0.561
Young age	−0.427	−0.56	−0.29	−0.429	−0.553	−0.305	−0.381	−0.509	−0.256	−0.384	−0.502	−0.267
NO_2_	0.026	0.006	0.045	0.011	−0.004	0.026	0.024	0.006	0.041	0.010	−0.004	0.023
Minimum temperature	0.562	0.206	0.918	0.034	−0.259	0.326	0.555	0.232	0.877	0.044	−0.234	0.323
Green space density	−0.860	−1.396	−0.328	−0.492	−0.944	−0.041	−0.781	−1.278	−0.291	−0.342	−0.795	0.108
	Respiratory disease mortality (10,682 cases)						Cardiovascular disease mortality (9969 cases)					
	Univariable			Multivariable (∆DIC = −32.1)			Univariable			Multivariable (∆DIC = −36.4)		
	Mean	Lower	Upper	Mean	Lower	Upper	Mean	Lower	Upper	Mean	Lower	Upper
Intercept				−9.09	−17.18	−1.042				−6.416	−12.344	−0.500
Indigenous degree	0.191	0.023	0.360	0.115	−0.046	0.279	0.134	−0.011	0.280			
Family resilience	−0.190	−0.370	−0.011	−0.049	−0.214	0.119	−0.135	−0.286	0.016			
Individual productivity	−0.243	−0.412	−0.074	−0.227	−0.387	−0.067	−0.125	−0.275	0.026			
Populous grassroots	0.432	0.273	0.596	0.347	0.191	0.508	0.442	0.307	0.582	0.411	0.281	0.545
Young age	−0.400	−0.555	−0.247	−0.404	−0.551	−0.260	−0.373	−0.507	−0.242	−0.384	−0.510	−0.260
NO_2_	0.029	0.013	0.046	0.013	−0.004	0.031	0.025	0.011	0.040	0.010	−0.004	0.025
Minimum temperature	0.482	0.158	0.810	−0.073	−0.320	0.467	0.389	0.055	0.726	−0.048	−0.336	0.240
Green space density	−1.058	−1.598	−0.525	−0.552	−0.836	−0.268	−0.883	−1.395	−0.379	−0.592	−1.045	−0.141

Note: Variables of the annual average of PM_10_, PM_2_._5_, O_3_, SO_2_, rainfall, dew point temperature, and relative humidity were not significant in any of the univariable models. ∆DIC indicates the difference of DIC between the multivariable model and the corresponding model with the intercept, offset term, and random effects. Table cells shaded in grey indicate that the association was statistically significant.

## Data Availability

The data are not publicly available due to restrictions of the Census and Statistics Department of the Hong Kong SAR Government.
